# The DRF motif of CXCR6 as chemokine receptor adaptation to adhesion

**DOI:** 10.1371/journal.pone.0173486

**Published:** 2017-03-07

**Authors:** Andrea Koenen, Aaron Babendreyer, Julian Schumacher, Tobias Pasqualon, Nicole Schwarz, Anke Seifert, Xavier Deupi, Andreas Ludwig, Daniela Dreymueller

**Affiliations:** 1 Institute of Pharmacology and Toxicology, Medical Faculty, RWTH Aachen University, Aachen, Germany; 2 Institute of Molecular and Cellular Anatomy, Medical Faculty, RWTH Aachen University, Aachen, Germany; 3 Laboratory of Biomolecular Research and Condensed Matter Theory Group, Paul Scherrer Institute, Villigen, Switzerland; Rutgers University, UNITED STATES

## Abstract

The CXC-chemokine receptor 6 (CXCR6) is a class A GTP-binding protein-coupled receptor (GPCRs) that mediates adhesion of leukocytes by interacting with the transmembrane cell surface-expressed chemokine ligand 16 (CXCL16), and also regulates leukocyte migration by interacting with the soluble shed variant of CXCL16. In contrast to virtually all other chemokine receptors with chemotactic activity, CXCR6 carries a DRF motif instead of the typical DRY motif as a key element in receptor activation and G protein coupling. In this work, modeling analyses revealed that the phenylalanine F^3.51^ in CXCR6 might have impact on intramolecular interactions including hydrogen bonds by this possibly changing receptor function. Initial investigations with embryonic kidney HEK293 cells and further studies with monocytic THP-1 cells showed that mutation of DRF into DRY does not influence ligand binding, receptor internalization, receptor recycling, and protein kinase B (AKT) signaling. Adhesion was slightly decreased in a time-dependent manner. However, CXCL16-induced calcium signaling and migration were increased. *Vice versa*, when the DRY motif of the related receptor CX_3_CR1 was mutated into DRF the migratory response towards CX_3_CL1 was diminished, indicating that the presence of a DRF motif generally impairs chemotaxis in chemokine receptors. Transmembrane and soluble CXCL16 play divergent roles in homeostasis, inflammation, and cancer, which can be beneficial or detrimental. Therefore, the DRF motif of CXCR6 may display a receptor adaptation allowing adhesion and cell retention by transmembrane CXCL16 but reducing the chemotactic response to soluble CXCL16. This adaptation may avoid permanent or uncontrolled recruitment of inflammatory cells as well as cancer metastasis.

## Introduction

Specific interactions between chemokines and their receptors regulate the sequential steps of diapedesis including adhesion and directional cell migration during inflammatory processes, tissue development, homeostasis, and cancer progression [1, 2]. CXCR6, first described as STRL33/BONZO [3], is expressed on different T cell subsets, macrophages, natural killer T (NK T) cells, fibroblasts and smooth muscle cells and is one of the T cell entry coreceptor used by HIV-1 [4–7]. The chemokine CXCL16, also referred to as “scavenger receptor for phosphatidylserine and low-density lipoprotein” (SR-PSOX), is the only known ligand of CXCR6 and is mainly expressed on endothelial cells [8, 9]. Together with CX_3_CL1, which binds to CX_3_CR1, CXCL16 is unique within the family of chemokines as it exists as a transmembrane and a soluble form [10–12], possibly acting as both adhesion and chemotactic molecule [4, 8, 13–17]. As a chemokine receptor, CXCR6 belongs to the class A of GPCRs. Upon activation, the receptor catalyzes the exchange of GDP to GTP in intracellular G_i_ proteins leading to the activation of phospholipase C, increase in inositol triphosphate concentration, and transient changes in intracellular calcium levels. In addition, activation of CXCR6 also results in the phosphorylation of signaling kinases such as protein kinase B (AKT). Activation of these signaling cascades induces cell migration, adhesion, proliferation, and survival [18].

The highly conserved aspartate-arginine-tyrosine (DRY) motif, located at the cytoplasmic side of transmembrane helix 3 (TM3) of most class A GPCRs, is a key motif for stabilizing the active state of the receptor and to activate G proteins, thereby regulating receptor activity [19–21]. Specifically, the negatively charged D^3.49^ (the number in superscript represents the position of the residue in the sequence according to the generic GPCRdb numbering [22]) forms a salt bridge with the positively charged R^3.50^ which keeps this arginine warped in an inactive conformation. Therefore, D^3.49^ has been shown to be involved in regulating the activity of many GPCRs including the chemokine receptors CXCR1, CXCR2, and chemokine (C-C motif) receptor 5 CCR5 [19–21]. Upon receptor activation, R^3.50^ is released from its interaction with D^3.49^ and extends to interact with Y^5.58^ and Y^7.53^, stabilizing the active state and building the binding pocket for the Gα-subunit of the G protein [23, 24]. The function of Y^3.51^ is not yet fully understood [25–27]. Mutation of this residue in CCR2 resulted in reduced activation [26], whereas in CXCR4 no effect was observed [27]. A recent study reported a relevance of this residue in G protein selectivity in Jurkat cells and HEK293T cells mediating cell specific functions [25]. While in human class A GPCRs position 3.51 predominantly holds a tyrosine residue (Y; ~66%, thus being the least conserved residue in the DRY motif), ~11% of receptors have a phenylalanine residue (F) in this position. For instance, in contrast to all other chemokine receptors with chemotactic activity, CXCR6 carries a F at position 3.51, resulting in an aspartate-arginine-phenylalanine (DRF) motif instead of the common DRY motif. This change is not only found in humans, but also in mouse, rat, and cattle [28], suggesting a special functional adaptation of the receptor.

In order to elucidate the functional significance of the DRF motif, receptor variants of CXCR6 including either the DRF or the DRY motif were expressed in embryonic kidney HEK293 cells and monocytic THP-1 cells and analyzed for ligand binding, internalization, receptor recycling, calcium signaling, AKT phosphorylation, cell adhesion, and cell migration. We found that converting the DRF to a DRY motif did not affect ligand binding or AKT signaling and reduced adhesion slightly in a time-dependent manner. In contrast, the DRF phenotype presented reduced calcium signaling capacity and chemotactic response in comparison to the DRY variant. These findings were confirmed by converting the DRY motif of the related receptor CX_3_CR1 into DRF. We thus conclude that the DRF motif in CXCR6 is an adaptation of the receptor that preserves its adhesion capacity in leukocyte subsets while diminishing its chemotactic function.

## Materials and methods

### Antibodies, chemokines and Fc-constructs

Recombinant human CXCL16 was purchased from PeproTech GmbH (Hamburg, Germany). Recombinant human and murine CX_3_CL1 were from R&D systems (Wiesbaden-Nordenstadt, Germany). Phycoerythrin (PE)-labeled monoclonal anti-CXCR6 (clone 56811) and isotype control (clone 133303) were from R&D Systems. Rabbit monoclonal anti-AKT (clone C67E7) and rabbit monoclonal anti-phospho-AKT (site Ser473, clone D9E) were from Cell Signaling Technology Inc. (New England Biolabs GmbH, Frankfurt a.M., Germany). Horse-radish peroxidase (HRP)-coupled anti-rabbit IgG and allophycocyanin (APC)-labeled polyclonal goat anti-human-Fc gamma were purchased from Jackson ImmunoResearch Europe Ltd (Suffolk, United Kingdom). Human CXCL16-Fc and CX_3_CL1-Fc-constructs, consisting of the extracellular cytokine domain C-terminally fused to the Fc-part of human IgG1a, were produced as described before [29].

### Modeling of CXCR6

The sequences of human CXCR6 and human CCR5 were aligned using Clustal Omega [30]. This initial alignment was manually refined using Chimera [31] to adjust some of the gaps in the loop regions. Using this alignment, CXCR6 was modeled with Modeller [32] using the structure of CCR5 (PDBid: 4MBS) as a template (residues 19–223,227–313) [33]. Residues missing in the template were refined using the loop optimization method in Modeller, and a disulfide bridge was added between residues Cys102 and Cys180. All models were subjected to 300 iterations of variable target function method optimization, thorough molecular dynamics and simulated annealing optimization and scored using the discrete optimized protein energy potential. The 20 best-scoring models were analyzed visually, and a suitable model (in terms of low score and structure of the loops) was selected. In addition to the crystallographic waters resolved within the transmembrane bundle in the structure of CCR5, we added additional ordered water molecules in this region as present in the high-resolution structure of the adenosine A2A receptor [34]. The protonation states of titratable groups were calculated using PROPKA [35] at pH 7.0 as implemented in PDB2PQR [36] to optimize the hydrogen bond network. The CHARMM-GUI Membrane Builder [36] was used to embed the model of CXCR6 in an explicit lipid bilayer composed by 117 1-palmitoyl-2-oleoyl-sn-glycero-3-phosphocholine (POPC) molecules, which was hydrated with a layer of water of ~35 Å on each side. Sodium and chloride ions were added to a concentration of 150 mM NaCl, and then additional ions were added to achieve charge neutrality. The dimensions of the final tetragonal box were approximately 73x73x108 Å, containing a total of ~54.000 atoms. The system was equilibrated using the protocol from the CHARMM-GUI Membrane [37] and then subjected to 50 ns of unrestrained molecular dynamics. Simulations were carried out with NAMD 2.10 [38] with the c36 CHARMM force field [39] in the NPT ensemble, using Langevin dynamics to control temperature at 300K, and at a time step of 2 fs while constraining all bonds between hydrogen and heavy atoms.

### Cell culture and cell preparation

THP-1 cells (human acute monocytic leukemia, DSMZ, Braunschweig, Germany) were cultured in RPMI1640 supplemented with 10% fetal bovine serum (FBS, PAN-Biotec GmbH, Aidenbach, Germany), and 1% penicillin streptomycin (P/S, Sigma-Aldrich, Darmstadt, Germany) by seeding at 2.0×10^5^ cells/ml and subculture at 1×10^6^ cells/ml. HEK293 cells (ATCC CRL-1573^TM^) were cultured in DMEM high glucose (Sigma-Aldrich) supplemented with 10% FBS and 1% P/S. Transfected and transduced cells were cultured in medium supplemented with additional 1 mg/ml geneticin (G418, Calbiochem EMD Chemicals, San Diego, USA) for selection. Prior to experiments, transduced cells were cultured without G418 for at least one week.

### DNA constructs

The DRF motif of human CXCR6 and the DRY motif of murine CX_3_CR1 were mutated to DRY and DRF, respectively, using QuikChange® (Agilent Technologies, Santa Clara, USA) site-directed mutagenesis of pcDNA3.1+ constructs of the receptors. For overexpression of CXCR6 and CX_3_CR1 variants, the respective cDNA was inserted in pLVX-IRES-Neo vectors (Clontech, Mountain View, CA, USA) using EcoRI and XbaI (CXCR6) or EcoRI and XhoI (CX_3_CR1).

### Lentiviral overexpression of CXCR6 and CX_3_CR1 variants

The lentivirus production and transduction of target cells were carried out as described previously [40].

### CXCR6/CX_3_CR1 cell surface expression and ligand binding

Paraformaldehyde (PFA)-fixed cells were incubated with 1 μg/ml PE-labeled anti-CXCR6 in PBS containing 0.2% bovine serum albumin (BSA) for 45 min and after washing subjected to fluorescence activated cell sorting (FACS) analysis (LSR Fortessa, Beckton Dickinson, Heidelberg, Germany). Receptor expression was measured as geometric mean of fluorescence intensity minus isotype control. Receptor expression and ligand binding of both CXCR6 and CX_3_CR1 were further investigated using chemokine-Fc-constructs. Fixed cells were resuspended in PBS/0.2% BSA. 1×10^5^ cells were incubated with 25 μl of 1:10 diluted Fc-construct on ice for 1 h, followed by 1 h incubation with APC-labeled anti-human-Fc gamma on ice for 1 h. Cells were subsequently subjected to FACS analysis. CXCL16-Fc binding was measured as geometric mean of fluorescence intensity in relation to background binding of anti-human-Fc gamma.

### Receptor internalization and recycling

HEK293 cells were harvested by accutase (Sigma-Aldrich) treatment and resuspended in PBS. Receptor internalization was induced by 15 min incubation with 20 nM soluble human CXCL16. To inhibit internalization, cells were pretreated for 15 min with 100 μM dynasore or 0.2% sodium azide (NaN_3_), or the cells were incubated on ice during the whole procedure. For measurement of receptor recycling, cells were washed with pre-warmed PBS (37°C) and resuspended in PBS. Subsequently, cells were placed on ice, washed once with ice-cold PBS and fixed with 1% PFA. Receptor surface expression was investigated by antibody staining as described above. THP-1 cells were harvested and resuspended in PBS. Receptor internalization was induced by 15 min incubation with 1 nM CXCL16-Fc fusion protein at 37°C. As “untreated” control cells, cells were kept on ice during the preincubation as well as the complete recycling step. After internalization, cells were washed twice with PBS and kept at 37°C in PBS for the indicated time. Subsequently, cells were transferred on ice to stop the recycling process. Receptor expression of CXCR6 was investigated using CXCL16-Fc fusion protein as described above.

### Proliferation assay

10,000 THP-1 or 5,000 HEK293 cells expressing CXCR6 variants were seeded in 100 μl cell culture medium supplemented with 10% FBS in 96-well plates. Cells were stimulated with recombinant human CXCL16 as indicated or left untreated as control. Cell proliferation was measured as increase of cell density by life-cell imaging every 2 h using the IncuCyte^TM^ Zoom (Essen Biosciences, Hertfordshire, UK). For statistical analysis, the fold increase in density within 72 h was calculated using the IncuCyte^TM^ software (Version 2015A, Essen Biosciences, Hertfordshire, UK). To control proliferation of THP-1 cells by an additional assay method, a commercial BrdU Cell Proliferation Chemiluminescent Assay Kit from Cell Signaling Technology was used. BrdU was added 24 h before measurement based on the proliferation kinetics observed in life- cell imaging.

### Adhesion assay

48-well cell culture plates were coated with 2 μg/ml anti-human Fc in PBS (120 μl/well) overnight at 4°C followed by 1 h blocking with PBS/1% BSA at room temperature (RT). Wells were coated with chemokine-Fc-constructs diluted 1:20 in PBS/1%BSA for 1 h at RT. THP-1 cells were harvested and stained with 1 μM calcein-acetoxymethylester (calcein-AM) for 15 min in PBS. After washing, 1×10^5^ cells in 200 μl HBSS were transferred to each well. Following sedimentation for 1 min at 300 g, plates were incubated at 37°C for the indicated time. After three washing steps with PBS, the number of remaining cells was determined by measuring the fluorescence intensity using a calibration curve (1:2 serial dilution of labeled cells). Fluorescence was measured using the FLUOstar OPTIMA plate reader (BMG LABTECH GmbH, Ortenberg, Germany) with an excitation wavelength of 488 nm and an emission wavelength of 526 nm.

### AKT activation

THP-1 cells were seeded in 6-well plates (1.5×10^6^ cells/well) in serum-free medium 16 h before stimulation. Cells were stimulated as indicated. Subsequently, cells were kept on ice. After centrifugation for 10 min at 300 g, cells were lyzed with 200 μl lysis buffer containing 20mM Tris, 150mM NaCl, 5mM EDTA, 30mM NaF, 5mM DTT, 1mM PMSF, 10mM pNPP, 1mM benzamidine, 10mM glycerophosphate, 1mM Na_3_PO_4_, 1% Triton X-100, and protease inhibitor mix (Complete, Roche Diagnostics, Germany) on ice for 30 min. After centrifugation for 10 min at 16,000 g, 25 μl of cell lysates were supplemented with 1x Laemmli buffer and subjected to SDS-PAGE and Western blotting. Membranes were blocked with TBST/3%BSA for 1 h and first probed for phosphorylated AKT, followed by total AKT determination. Antibodies were used according to the manufacturer’s protocol. The HRP-coupled secondary antibody was applied in TBST for 1 h at RT. HRP-mediated chemiluminescence (ECL Plus HRP substrate solution, Amersham, Little Chalfont, UK) was measured using the LAS-3000 (Fujifilm, Dusseldorf, Germany), and signals were quantified using Aida Image Software (Elysia-raytest GmbH, Straubenhardt, Germany).

### Calcium assay

THP-1 cells were labeled for 15 min with 2 nM Fluo4-AM (Life Technologies, New York, USA) in HBSS supplemented with 10 mM HEPES, 710 ng/ml Probenicid, and 2 nM Pluronic F-127. Cells were washed twice and resuspended at a density of 2×10^6^ cells/ml in HBSS supplemented with 10 mM HEPES and 710 ng/ml Probenicid. 100 μl of the cell suspension were added to a black 96-well-plate with a clear bottom. Calcium-influx upon stimulation with CXCL16 and CCL2 was measured at 37°C in the FLUOstar OPTIMA plate-reader. After baseline measurement for 10 s, CCL2 (3 nM final, positive control) or soluble CXCL16 (1-20 nM CXCL16 final) were injected. As buffer control, 20 μl of calcium-assay buffer containing 0.5% BSA was injected. Equal loading was controlled by injecting Triton X-100 (0.1% final, Sigma-Aldrich), background fluorescence was determined by further addition of 15 mM ethylene glycol tetraacetic acid (EGTA). No differences between cells expressing different variants of CXCR6 were observed. The fluorescence was constantly measured at an excitation wavelength of 488 nm and an emission wavelength of 526 nm.

### Chemotaxis assay

For THP-1 chemotaxis assays, a Boyden chamber (Neuroprobe, Gaithersburg, MD) and polycarbonate filter membranes with 8 μm pores were used (Neuroprobe, Gaithersburg, MD). Lower chambers were filled with stimulus solution (CXCL16, CX_3_CL1, or CCL2, respectively, at the indicated concentrations in assay buffer (RPMI/0.2% BSA)). 6×10^4^ THP-1 cells were added to the upper chamber. After incubation for 2 h, migrated cells were quantified in the lower chamber by measurement of endogenous glucuronidase activity as described [29] and quantified as number of migrated cells in the presence of chemotactic stimulus in relation to random migration (absence of chemotactic stimulus).

### Statistics

Quantitative data are shown as mean + standard deviation (SD) calculated from at least three independent experiments and transductions. Statistics were calculated using PRISM5.0 (GraphPad Software, La Jolla, CA) and JMP 10.0 (SAS Institute GmbH, Cary North Carolina, USA) and tested for outliers. Statistical analyses of AKT activation and chemotaxis experiments were performed with raw data (box-cox transformed in case of AKT activation), but are shown as normalized data for better visualization. Differences between receptor variants in migration experiments were analyzed by determination of the area under the curve (AUC). Used statistic tests are indicated within the figure legends. In case of multiple comparisons, p-values were corrected by false discovery rate (FDR).

## Results

### Environment of F^3.51^ of the DRF motif in the CXCR6

**Fig 1:**
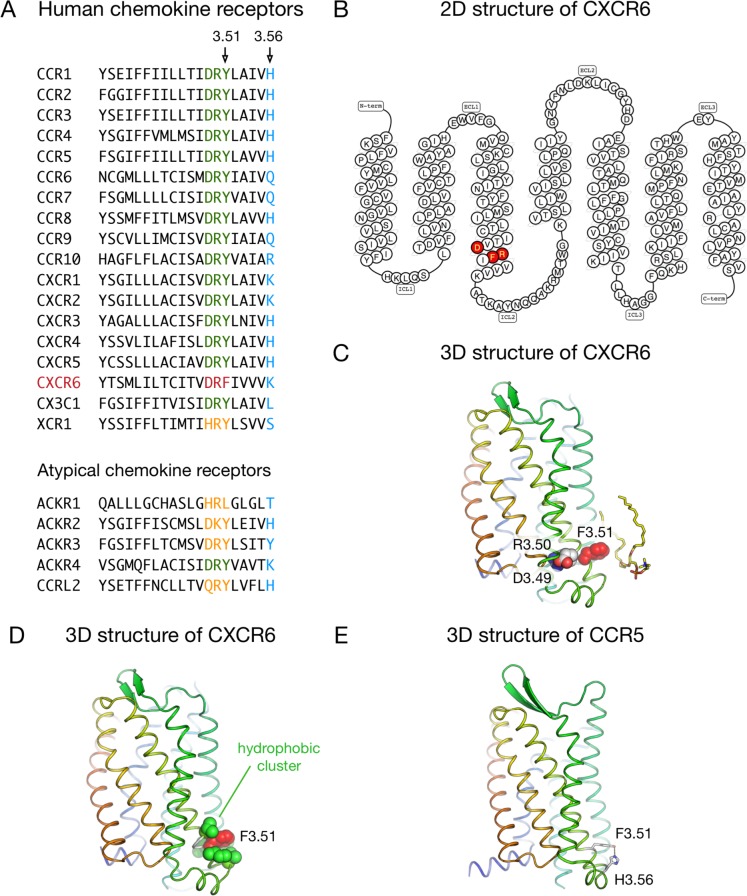
Sequences and structural features of CXCR6 around the DRF motif. A: Sequence of TM3 (residues 3.37–3.56) in human chemokine receptors. The DRY motif (residues 3.49–3.51) is highlighted in green, the DRF motif of CXCR6 in red, other motifs at this position in orange, and residues at position 3.56 in blue. B: 2D structure of the human CXCR6 (obtained from the GPCRdb [67]), with DRF motif highlighted in red. C/D: Snapshot from the molecular dynamics simulation of the 3D model of the human CXCR6. The residues in the DRF motif (D126^3.49^, R127^3.50^ and F128^3.51^) are shown as spheres, and a lipid molecule in contact with F128^3.51^ is shown as sticks (C). The cluster of hydrophobic residues around F128^3.51^ is shown in D. The rest of protein side chains, lipids, water molecules, and ions are not displayed for clarity. E: 3D structure of the human CCR5 receptor, showing the hydrogen bond between residues at positions 3.51 and 3.56.

CXCR6 is the only member of the G protein-coupled chemokine receptors that carries an F amino acid at position 128^3.51^, resulting in a DRF motif instead of the much more common DRY motif at the cytoplasmic end of TM3 (Fig 1A and 1B). In order to analyze the possible structural roles of this DRF motif, we built a three-dimensional model of the CXCR6 receptor and run a molecular dynamics simulation to assess the interactions of F^3.51^ with its environment. We observed that F^3.51^ is located at the surface of the receptor and exposed to the interface between the lipid bilayer and the cytoplasmic milieu where it can interact with phospholipid head groups (Fig 1C). In addition, F^3.51^ is surrounded by a cluster of hydrophobic residues from TM3 (V^3.48^, I^3.52^ and V^3.55^) and TM5 (V^5.60^ and I^5.61^) in our CXCR6 model (Fig 1D). We further questioned for the pattern of interaction at this position in receptors that hold the most common Y^3.51^. Inspection of the crystal structures of peptide/protein receptors revealed a conserved hydrogen bond donor/acceptor residue about one turn (five residues) after Y^3.51^ that can interact with this residue. For instance, in the chemokine receptor CCR5 Y^3.51^ is interacting with histidine^3.56^ (Fig 1E). In CXCR6, however, F^3.51^ cannot hydrogen bond lysine^3.56^. This may alter the local structure/dynamics properties at this region, thereby affecting receptor activity.

### Rescue of the DRY motif in the CXCR6 does not affect ligand binding, receptor internalization or recycling

**Fig 2:**
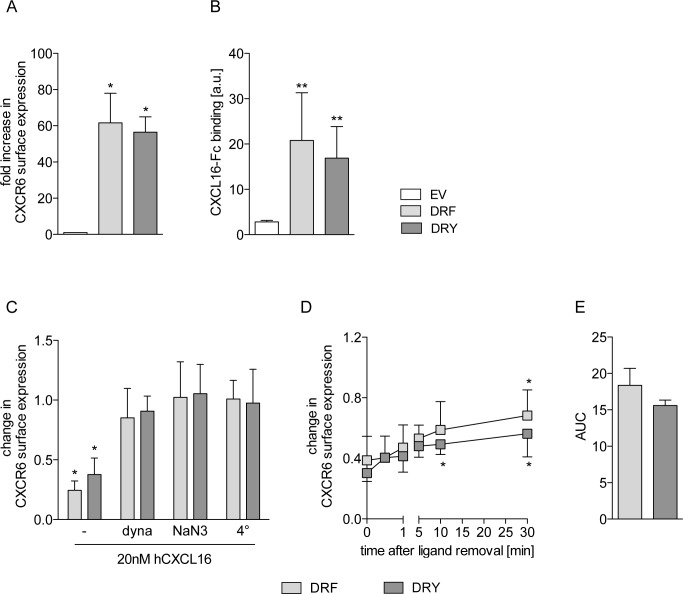
Rescue of the DRY motif in CXCR6 does not affect ligand binding or receptor recycling in HEK293 cells. HEK293 cells were transduced with lentivirus encoding the human CXCR6 variants or the EV control. A/C-E: Surface expression of CXCR6 in untreated cells (A, n = 4), after receptor internalization induced by 15 min treatment with soluble CXCL16 (C, n = 3), and receptor recycling (D, dyna = dynasore, n = 4) was determined by FACS analysis using an antibody against human CXCR6. Signals were expressed in relation to control (EV in A, untreated cells in C/D). Receptor recycling was further quantified as area under the curve of data shown in D (AUC, E). B: Ligand binding was analyzed by incubation with CXCL16-Fc fusion protein and FACS analysis (n ≥ 3, a.u. = arbitrary units). Statistical differences were analyzed by one-sample t-test (A and C, hypothetical value 1) or Student’s t-test (B, D and E). Asterisks indicate differences to control (*p<0.05, **p<0.01).

**Fig 3:**
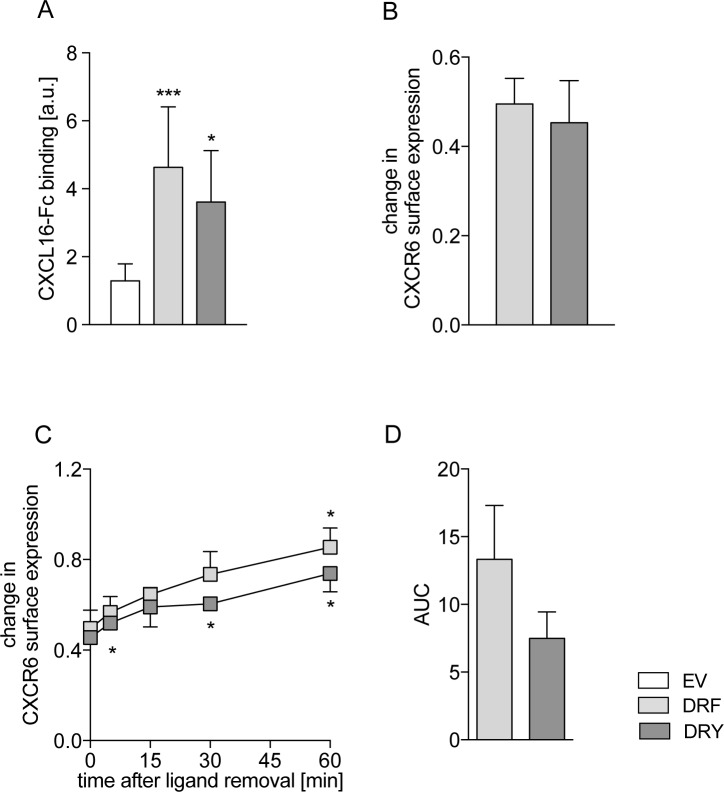
Rescue of the DRY motif in CXCR6 does not affect ligand binding or receptor recycling in THP-1 cells. THP-1 cells were transduced with lentivirus encoding human CXCR6 variants or EV control. A: Ligand binding of the CXCL16-Fc fusion protein (n ≥ 6, a.u. arbitrary units). B-D: Surface expression of CXCR6 after receptor internalization (B, 1 nM CXCL16-Fc for 15 min at 37°C), and receptor recycling (C, 1 nM CXCL16-Fc for 15 min at 37°C and subsequent removal) was analyzed by incubation with CXCL16-Fc fusion protein and FACS analysis (n = 3). Recycling was determined after 0, 5, 15, 30, and 60 min. Signals were expressed in relation to untreated cells (1 nM CXCL16-Fc for 15 min at 4°C). Receptor recycling was further quantified as area under the curve of data shown in C (AUC, D). Statistical differences were analyzed by one-sample t-test (B, hypothetical value 1) or Student’s t-test (A, C and D). Asterisks indicate differences to control (*p<0.05, ***p<0.001).

To assess the functional role of the endogenous DRF motif of CXCR6, receptors featuring either DRF or DRY (CXCR6[DRF] or CXCR6[DRY]) were expressed in human cell lines that do not express detectable levels of endogenous CXCR6 (S1A, S1B and S2A Figs). Ligand binding and receptor internalization are the first steps in chemokine receptor signaling [41]. To investigate these processes, CXCR6 variants or an empty vector (EV, as control) were expressed in HEK293 cells. Expression of the CXCR6 variants did not influence basic cell functions like proliferation (S1C Fig). FACS analysis using an antibody against human CXCR6 revealed that both CXCR6[DRF] and CXCR6[DRY] were expressed at a similar level on the cell surface (Fig 2A, S1A Fig). We also found that the levels of ligand binding to CXCR6[DRF] and CXCR6[DRY], assessed by binding of a human CXCL16-Fc fusion protein [29], were very similar, whereas EV control cells displayed only background binding (Fig 2B, S1B Fig). Furthermore, neither receptor variant differed in ligand-induced reduction of cell surface expression (Fig 2C, first two bars). This decrease was suppressed by the dynamin inhibitor dynasore, by sodium azide and at low temperature (4°C), indicating that both receptor variants undergo ligand-induced internalization via the endocytotic pathway (Fig 2C). Finally, both receptor variants showed significant levels of re-expression on the cell surface 30 min after removal of the soluble ligand (Fig 2D), which did not differ between the variants (Fig 2E), indicating similar levels of receptor recycling. The CXCR6-CXCL16 axis plays essential roles in the adhesion and chemotactic recruitment of leukocytic cells. To study CXCR6 variants in leukocytic cells we used the monocytic cell line THP-1. As observed in HEK293 cells, the two variants were equally expressed on the cell surface of THP-1 cells and did not differ in ligand binding ability (Fig 3A, S2A Fig). Neither CXCR6 expression nor stimulation with CXCL16 influenced cell proliferation in any of the two variants (S2B and S2C Fig). Furthermore, CXCR6[DRF] and CXCR6[DRY] expressing THP-1 cells did not differ in ligand-induced downregulation of the receptor from the cell surface (Fig 3B) and its re-expression on the cell surface after removal of the soluble ligand (Fig 3C and 3D). This indicates a similar internalization and recycling of CXCR6[DRF] and CXCR6[DRY] also in THP-1 cells.

### Rescue of the DRY motif in the CXCR6 has no major impact on AKT signaling and adhesion

**Fig 4:**
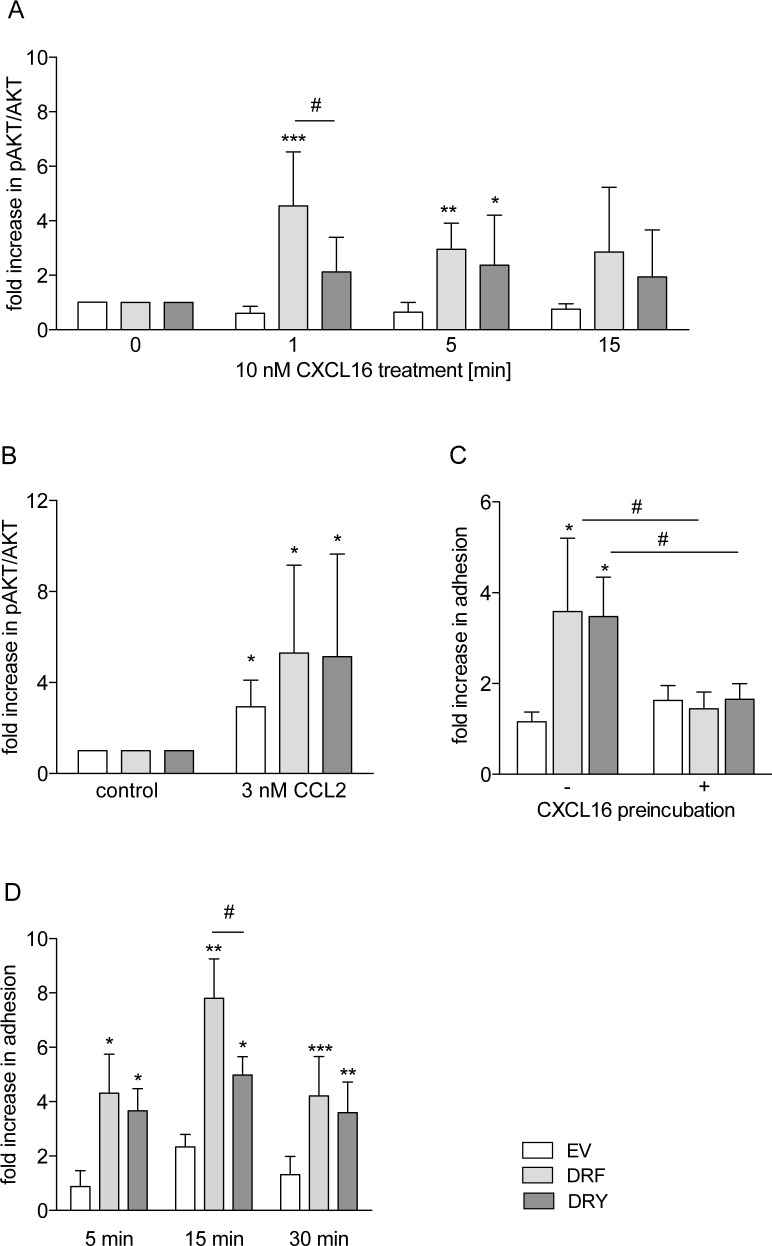
Rescue of the DRY motif in CXCR6 has no major impact on AKT signaling and adhesion. THP-1 cells were transduced with lentivirus encoding human CXCR6 variants or EV control. A/B: AKT activation upon stimulation with 10 nM soluble CXCL16 (A, n = 7) or with 3 nM CCL2 (B, 10 min, control of general responsiveness, n = 7) was measured as ratio of phosphorylated AKT (pAKT) to total AKT investigated by Western blot analysis. Data were expressed in relation to buffer control stimulated cells. C: Adhesion to immobilized CXCL16-Fc for 30 min was normalized to cells adhering to anti‑human-Fc (set = 1 for each cell-type, n = 4). Cells were pretreated with 100 nM soluble CXCL16 for 15 min to indicate specificity of CXCL16-CXCR6-mediated adhesion. D: Adhesion to immobilized CXCL16-Fc for 5, 15, and 30 min was normalized to cells adhering to anti‑human-Fc (set = 1 for each cell-type, n = 3). Statistical differences were analyzed by one-sample t-test (C for differences to adhesion to anti-human-Fc, hypothetical value 1) or Student’s t-test (A-D, with Welch’s correction in A). Asterisks indicate differences to control (untreated cells in B/C, EV in A/D), hashes indicate differences between receptor variants in A and D and CXCL16 preincubation in C (*/#p<0.05, **p<0.01, ***p<0.001).

We also investigated whether the presence of DRF instead of DRY could be related to changes in the phosphorylation of AKT, which was reported to be a downstream event of CXCR6 signaling [18]. We observed that both CXCR6[DRF] and CXCR6[DRY] induced AKT signaling after 1 and 5 minutes of stimulation with CXCL16, whereas EV control cells did not respond (Fig 4A, S2D Fig). Stimulation with CCL2 as control resulted in comparable levels of AKT activation in all three THP-1 cell lines (Fig 4B). CXCR6[DRF] expressing cells showed a slightly higher AKT activation after 1 min of CXCL16 stimulation compared to CXCR6[DRY] expressing cells. Besides this small increase we did not observe differences in the overall pattern of AKT activation between the two receptor variants when cells were stimulated with CXCL16.

Since the transmembrane form of CXCL16 mediates cell adhesion of CXCR6-expressing leukocytes [42–44], we finally investigated the possible influence of the DRF motif on THP-1 cell adhesion. While background adhesion of THP-1 cells to anti-human-Fc was negligible (S2E Fig), adhesion to immobilized CXCL16-Fc fusion protein was approximately 4-fold increased in cells expressing CXCR6[DRF] or CXCR6[DRY]. This adhesion was completely blocked by preincubation with soluble CXCL16, confirming that the interaction of immobilized CXCL16 with its receptor is sufficient to mediate cell adhesion. This specific CXCL16-CXCR6-interaction did not differ between the two receptor variants (Fig 4C). Although the overall pattern of adhesion as well as detachment of bound cells by repeated rigorous washing were similar in the receptor variants expressing cells between 5 and 30 minutes, CXCR6[DRF] expressing cells showed a 2-fold higher increase of adhesion at 15 minutes compared to CXCR6[DRY] expressing cells (Fig 4D).

### The DRF motif is associated with impaired calcium signaling and migratory potential of CXCR6

**Fig 5:**
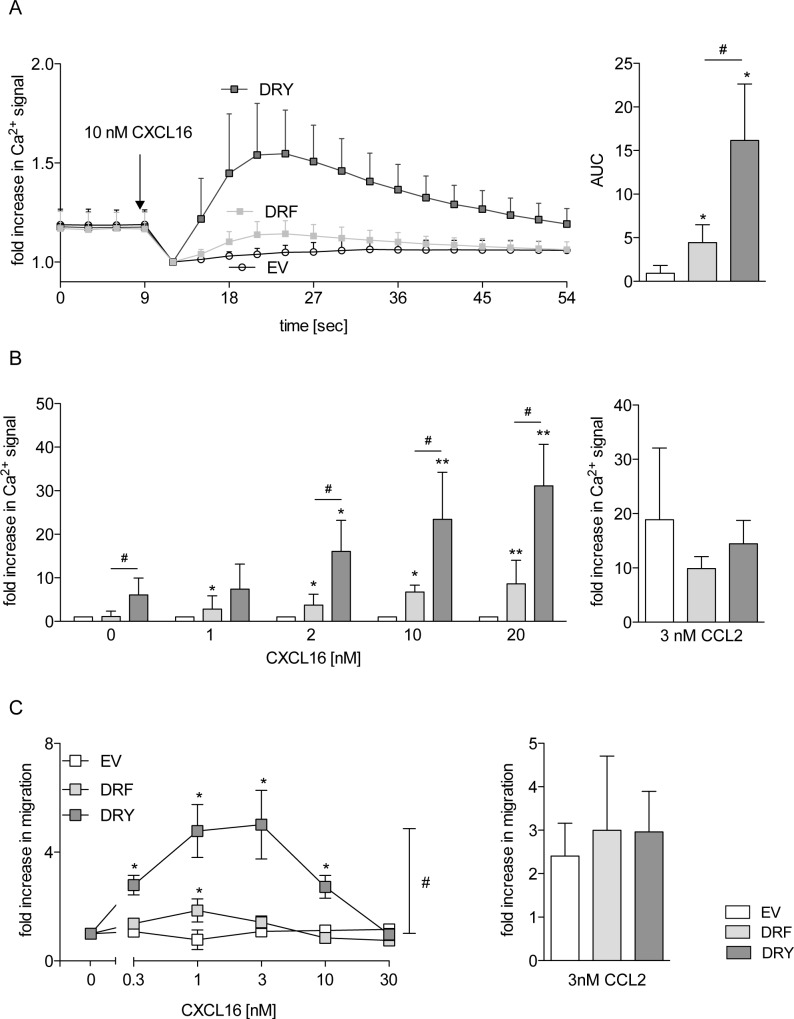
The DRY motif is optimized for calcium signaling and chemotaxis. THP-1 cells were transduced with lentivirus encoding human CXCR6 variants or EV control. A/B: Cells were loaded with Fluo4-AM and stimulated with 10 nM soluble CXCL16 (A, n = 4), increasing concentrations of soluble CXCL16 (n = 4), or 3 nM CCL2 (control of general responsiveness, n = 3) (B). The increase of the Ca^2+^ signal was measured as increase in Fluo4-AM-fluorescence. Data were normalized to signals at 9 sec in A and quantified as AUC. In B, maximal fluorescence intensity was determined, and minimal fluorescence intensity was subtracted for each calcium response and expressed in relation to the response of EV cells for each concentration. C: Chemotaxis against increasing concentrations of soluble CXCL16 or 3 nM CCL2 (control of general migratory potential, n ≥ 4) was analyzed in a Boyden chamber assay (n ≥ 4). Statistical differences were analyzed by Student’s t-test. Asterisks indicate differences to control (EV in A, each buffer control in B, random migration in C), hashes indicate differences between receptor variants (*/#p<0.05, **p<0.01).

We also assessed the potential role of the DRF motif on the transient increase in intracellular calcium concentration upon receptor activation. We observed that THP-1 cells expressing CXCR6[DRF] showed a 5-fold increase in the calcium response upon CXCL16 stimulation with respect to EV cells. This response was significantly higher for CXCR6[DRY] (3-fold higher than for CXCR6[DRF], Fig 5A). We also observed that increasing concentrations of CXCL16 resulted in a higher calcium response in cells expressing CXCR6[DRY], whereas expression of CXCR6[DRF] resulted in a constant low response (Fig 5B, left panel). The calcium response due to other endogenous chemokine receptors was not changed as indicated by similar levels of the calcium response upon CCL2 stimulation of THP-1 cells expressing the two CXCR6 variants (Fig 5B, right panel) or EV.

Calcium signaling is required for chemokine-induced cell migration [45]. Therefore, we investigated if the observed differences in calcium response would influence cell migration towards soluble CXCL16. Random migration (migration of cells without chemotactic stimulus) and CCL2-induced chemotaxis of THP-1 cells were not changed by expression of neither of the two CXCR6 variants and were not different from the EV control (Fig 5C right panel, S2F Fig). EV control cells did not migrate towards CXCL16, and cells expressing CXCR6[DRF] showed only weak migration in response to 1 nM CXCL16. In contrast, cells expressing CXCR6[DRY] clearly showed a concentration dependent CXCL16-induced chemotaxis with typical dose response curve with an optimum between 1 and 3 nM CXCL16 (Fig 5C, left panel).

### The presence of a DRF motif generally impairs for chemotaxis

**Fig 6:**
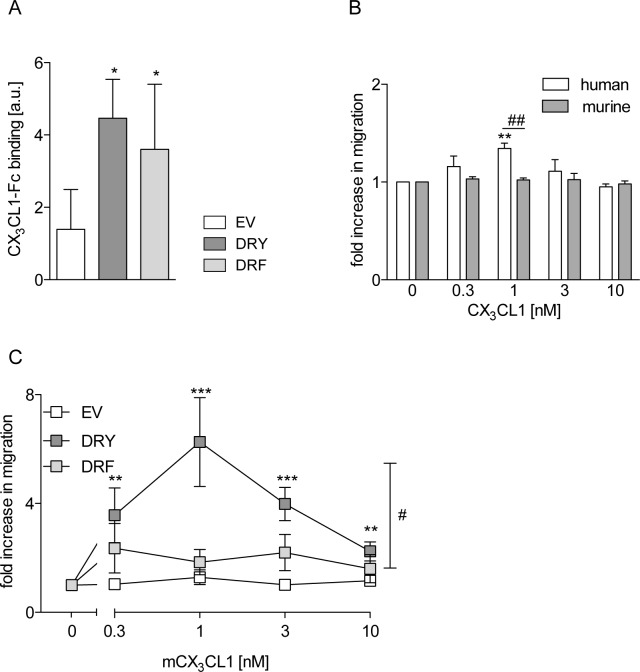
The DRF motif impairs chemotaxis. THP-1 cells were transduced with lentivirus encoding murine CX_3_CR1 variants or EV control. A: Ligand binding was analyzed by incubation with human CX_3_CL1-Fc fusion protein and FACS analysis (n = 3). B/C: Chemotaxis against increasing concentrations of soluble CX_3_CL1 was analyzed in a Boyden chamber assay. In B, wild type cells were investigated for chemotaxis towards soluble murine or human CX_3_CL1 (n = 3), and in C cells overexpressing murine receptor variants were assayed for chemotaxis towards murine CX_3_CL1 (n ≥ 3). Statistical differences were analyzed by one-sample t-test (B, hypothetical value 1) or Student’s t-test (A-C). Asterisks indicate differences to control (EV in A, random migration in B/C), hashes indicate differences between receptor variants (*/#p<0.05, **/##p<0.01, ***p<0.001).

CX_3_CL1 is the second transmembrane chemokine and the only ligand for CX_3_CR1 which contains the typical DRY motif of G-protein-coupled chemokine receptors. To test the hypothesis that the presence of a DRF motif generally results in impaired chemotaxis, we mutated the DRY motif of CX_3_CR1 (CX_3_CR1[DRY]) to DRF (CX_3_CR1[DRF]). As THP-1 cells endogenously express CX_3_CR1, the murine receptor variants were used in this study. The murine CX_3_CR1 is capable of binding both human and murine CX_3_CL1, whereas human CX_3_CR1 cannot interact with murine CX_3_CL1. Ligand binding levels, assessed by binding of human CX_3_CL1-Fc fusion protein, did not differ between THP-1 cells expressing CX_3_CR1[DRY] or CX_3_CR1[DRF] (Fig 6A, S3 Fig). To verify that endogenous CX_3_CR1 expression may not influence the analysis of the chemotactic response, wild type cells were investigated for migration towards human and murine CX_3_CL1. Only human CX_3_CL1 elicited a migratory response (Fig 6B) of wild type THP-1 cells. Remarkably, the concentration dependent CX_3_CL1-induced chemotaxis, which was observed in cells expressing murine CX_3_CR1[DRY], was abrogated in cells expressing murine CX_3_CR1[DRF] and absent in EV control cells (Fig 6C). Thus, the DRF motif not only impairs the chemotactic response in CXCR6 but also in CX_3_CR1. These results support the hypothesis that the DRF motif of CXCR6 is specifically linked to the impaired chemotactic response of this receptor.

## Discussion

In the present study we showed that the presence of a DRF motif at the cytoplasmic end of TM3 in CXCR6 that is unique among chemokine receptors is associated with a reduced calcium signaling and migration potential but enhancing adhesion in a time-dependent manner without influencing ligand binding, receptor internalization, receptor recycling, and AKT signaling. Moreover, mutation of the endogenous DRY into DRF in CX_3_CR1 diminished the migratory response to CX_3_CL1. We hypothesize that the DRF motif is an adaptation of the CXCR6 towards its role in adhesion while reducing its chemotactic potential. This could indicate that the main function of this receptor is to mediate cell retention to tissue cells rather than cell attraction.

The endogenous ligand of the CXCR6, CXCL16, exists both as transmembrane and soluble forms, mediating firm cell adhesion and chemotaxis [4, 8, 13–17]. Both processes require the interaction of the ligand with its receptor. We have shown that the presence of the DRF motif does not influence ligand binding in HEK293 or THP-1 cells. The data from leukocytic cells are in line with results recently reported for CXCR6 variants that were overexpressed in Jurkat cells (immobilized human T lymphocytes) [25]. In contrast, the same study showed reduced binding of CXCL16 to CXCR6[DRY] in comparison to CXCR6[DRF] in HEK293T cells. This discrepancy is difficult to explain, but differences between HEK293 (used in this study) and HEK293T cells have been reported before, showing for example differences in genome dynamics [46]. Further, slight differences in the used assay procedures, especially regarding the used soluble CXCL16 (^125^I-CXCL16 and CXCL16-Fc), may result in different sensitivities to detect the modest changes in ligand binding observed in HEK293T cells [25].

Chemokine receptors are regulated by desensitization as well as by internalization of the ligand-receptor-complex, and both processes are critical for chemokine-induced cell functions such as cell migration [41]. Cells expressing CXCR6[DRF] or CXCR6[DRY] did not differ neither in internalization/recycling nor in the speed of these two processes. Inhibition of internalization by dynasore treatment indicated the involvement of dynamin-mediated formation of clathrin-coated pits or dynamin-dependent caveolae-mediated endocytosis [47, 48]. While lipid raft internalization could also contribute to CXCR6 recycling [41], this mechanism seems of minor importance as indicated by the complete inhibition with dynasore. Previous reports showed a proliferative effect of the CXCR6/CXCL16 axis by the activation of the PI3K/AKT and ERK pathways [5, 49]. In the present study we could not observe a stimulation of proliferation of HEK293 or THP-1 cells expressing the CXCR6 variants in the presence or the absence of CXCL16. This effect may vary between different cell types as the proliferative effect of CXCL16 was only observed in smooth muscle cells or glioma cells [25]. In contrast, high expression of CXCR6 and CXCL16 in mammary or liver cancers correlated with reduced proliferation and invasiveness [50, 51]. These cells may differ in the expression of involved G proteins and signaling pathways compared to the kidney or monocytic cells used in the present study. Furthermore, it has been shown that the proliferative effect of AKT activation varies greatly among immune cell subtypes [52]. Nevertheless, we observed that AKT signaling was not influenced by the mutation of DRF to DRY in CXCR6. Transmembrane CXCL16 has been shown to be important for adhesion of monocytes, platelets, and erythrocytes to the endothelium as well as of lymphocytes to the follicle-associated epithelium in the gut [8, 16, 17]. Furthermore, CXCL16/CXCR6 interaction was required for full NK T cell and dendritic cell activation and maturation [53], and also mediated the adhesion of cytotoxic T cells to tumor-associated macrophages (TAM) [50]. Our data in THP-1 cells expressing the CXCR6 variants confirmed the necessity of CXCR6 for adhesion to CXCL16. No overall differences between the DRF and DRY variants were observed assessing the whole time span from 5 to 30 minutes, which is in line with the equal ligand binding of these two variants. Similar results have been obtained for the CX_3_CR1/CX_3_CL1 interaction showing that CX_3_CR1 mediates adhesion to CX_3_CL1, but that the presence of a functional DRY motif is not required [40]. However, CXCR6[DRF] expressing cells showed a 2-fold higher adhesion after 15 minutes in comparison to CXCR6[DRY] expressing cells. As summarized and discussed later, this higher adhesion could favor retention of cells and limit harmful cell recruitment resulting from high concentrations of soluble CXCL16 within injured tissues.

Interaction of chemokine receptors with their ligands results in changes of the intracellular calcium level. While activation of CXCR6 with soluble CXCL16 resulted in increased calcium signaling, we observed that turning the DRF sequence into the more common DRY motif resulted in a higher calcium response. The structural role of residue Y^3.51^ in GPCR activation is unclear, and its mutation often affects receptor function only marginally. However, in particular cases, this residue is indeed functionally important [25–27]. For instance, mutation of the Y^3.51^ in the chemokine receptor CCR2 indicated that this residue is essential for activity [26]. Further, viral 7-TM receptor homologues showed increased activity when hydrophobic side chains like phenylalanine were replaced with polar side chains like tyrosine [54]. Thus, it seems that the presence of the polar hydroxyl group of the tyrosine plays a more important role in receptor activity than its aromatic nature. According to our model and in agreement with the available GPCR crystal structures, the DRF motif of CXCR6 is located at the receptor surface and in the interface between the lipid bilayer and the cytoplasm where it can interact with the polar phospholipid head groups and water molecules. These interactions would be weaker in receptors featuring F^3.51^ instead of Y^3.51^, which could be related to the lower levels of agonist-induced calcium production and migratory response in CXCR6. Indeed, it has been recently shown that the nature of the phospholipid head groups can affect the activity of the β2-adrenergic receptor [55]. In addition, in receptors that feature Y^3.51^ the hydroxyl group of this residue can establish a hydrogen bond to a basic residue at position 3.56 as shown for CCR5. This interaction is not possible in receptors containing F^3.51^, such as CXCR6 or PAR1 [56], which might also be related to the lower agonist-induced calcium production in CXCR6. Interestingly, it was recently shown that CXCR6[DRY] and CXCR6[DRF] do not differ in terms of intracellular calcium signaling when the receptor variants were overexpressed in Jurkat cells. Moreover, the same study showed that CXCR6-mediated signaling depends on the cell-specific use of G proteins [25]. Obviously, the mutation of DRF to DRY in CXCR6 is not relevant in Jurkat cells, while it clearly increases calcium signaling and cell migration in THP-1 cells. These differences may be caused by cell type biased signaling such as the use of different G proteins and could indicate a divergent relevance of the evolutionary change in CXCR6 in lymphocytic and monocytic cells.

One important function of soluble CXCL16 is the recruitment of CXCR6-expressing cells to the site of acute and chronic inflammation. For instance, CXCL16 released by irradiated breast cancer cells leads to the recruitment of effector T cells [57]. Also in the liver, the CXCL16/CXCR6 axis is required for the accumulation of NK T cells and the recruitment of macrophages in steatohepatitis in chronic hepatic injury [6, 15]. Our analysis of CXCL16-induced migration of CXCR6-expressing THP-1 cells confirmed the migratory function of soluble CXCL16. However, in comparison with CCL2-induced migration, the chemotaxis of CXCR6[DRF] cells towards CXCL16 was very weak. This is in line with a previous report showing that CXCR6 expressed in T cells of acutely or chronically inflamed lungs only mediates weak chemotaxis [4]. In contrast to CXCR6, CX_3_CR1, which carries a DRY motif, is fully capable of inducing chemotaxis as indicated by our observation that cells expressing CX_3_CR1 showed a chemotactic response to CX_3_CL1 that was similar to that induced by CCL2. We have shown that this response can be impaired by mutating the DRY motif of CX_3_CR1 to DRF, thus confirming the role of position 3.51 in chemotaxis. In summary, migration and calcium signaling, which are closely linked cellular processes [58], were both reduced in CXCR6[DRF] compared to CXCR6[DRY] cells. As the ligand binding affinity was not changed it seems very likely that the changes in the microenvironment of F^3.51^ described above led to reduced effectiveness in signal transduction towards the G protein in CXCR6.

THP-1, HEK293, and Jurkat cells, which were used in the present and other studies to assess the functional role of the endogenous DRF motif of CXCR6, are all cell lines. To further address the differential function of the DRF motif in adhesion and migration under more physiological conditions, primary cells should be used. However, a lot of primary cells, including different T cell subsets, macrophages, natural killer T (NK T) cells, fibroblasts and smooth muscle cells express CXCR6 endogenously [4–7], thus complicating the investigations of receptor variants. For this reason we chose murine bone marrow derived macrophages from CXCR6 knockout mice as target cells. However, we could not achieve sufficient transduction/transfection efficiency to perform functional assays. These limitations will make the analysis of CXCR6 variants in different primary cell types a challenging approach for future projects.

CXCR6 underwent an evolutionary change from the typical DRY motif, which is present in CXCR6 of the elephant shark, into a DRF motif found in vertebrates [28]. This change is unique in the chemokine receptor family, and we can only speculate about the reason of this change. The underlying need for this type of mutation in CXCR6 might be explained by the nature of its ligand, CXCL16. As discussed above, the transmembrane and the soluble forms of CXCL16 differ in their function within the body. Transmembrane CXCL16 is expressed on various cell types at interfaces including endothelial and epithelial cells and within tissues. In this scenario, it could be important that CXCR6 can mediate cell adhesion and retention. In addition, soluble CXCL16 is constitutively released within the tissue and into the blood at high levels. We propose that soluble CXCL16 can still induce migration to some degree, which may be relevant for recruitment and homing of CXCR6 expressing NK T cells or T cells [15, 53]. However, this function could be less vital since several other soluble chemokines can fulfill this function. In some cases the high levels of released chemokine could even be detrimental. High levels of CXCL16 are found in the lung of patients with sarcoidosis, or interstitial lung diseases [59]. Further, elevated serum levels of CXCL16 were found in patients with renal injury, acute coronary syndrome, atherosclerosis, or cardiac surgery [60–62]. In such situations the increased recruitment of cytotoxic T cells, T helper cells, and NK T cells would promote chronic inflammation and fibrosis [6, 15, 63, 64]. Moreover, in various cancers chemokines can induce metastasis via the induction of cell migration. CXCR6 is expressed on various cancer types concomitant with high levels of CXCL16 in tumors [50, 59, 65, 66]. The attenuation of CXCR6 induced migration could serve to limit these harmful effects. Furthermore, the cell retention of cytotoxic T cells on CXCL16 expressing tumors through the main function of the receptor in adhesion, which is not altered by the unusual DRF motif, could dampen cancer growth and metastasis [50, 66]. Thus, regarding the divergent roles of CXCL16 during homeostasis, inflammation, and cancer the DRF motif of CXCR6 may represent an adaptation of the receptor that allows cell adhesion and retention while avoiding permanent or uncontrolled recruitment of inflammatory cells as well as cancer metastasis.

## Supporting information

S1 Fig Supporting information HEK cells. HEK293 cells were transduced with lentivirus encoding human CXCR6 variants or EV control. A: Surface expression of CXCR6 was determined by FACS analysis using an antibody against human CXCR6. Representative histograms are shown. B: Ligand binding was determined by incubation with CXCL16-Fc fusion protein and FACS analysis. Representative histograms are shown. C: Cell proliferation was determined for 72 h by automated real time cell imaging. No statistic differences were observed (n = 3).
(TIF)

S2 Fig Supporting information THP-1 cells expressing CXCR6. THP-1 cells were transduced with lentivirus encoding human CXCR6 variants or EV control. A: Ligand binding of the CXCL16-Fc fusion protein. Representative histograms are shown. B/C: Cell proliferation in the presence or absence of soluble CXCL16 was determined for 72 h by automated real time cell imaging in B and BrdU assay in C (n = 3). Data in C were expressed in relation to cells in the absence of soluble CXCL16. D: AKT activation was investigated by Western blot analysis. Representative blots are shown. E: Adhesion to immobilized anti-human-Fc was investigated as control experiment (n = 4). F: Random migration was investigated in a Boyden chamber assay (n ≥ 4). No statistic differences were observed in B to E.
(TIF)

S3 Fig Supporting information THP-1 cells expressing CX_3_CR1. THP-1 cells were transduced with lentivirus encoding murine CX_3_CR1 variants or EV control. Ligand binding was analyzed by incubation with CX_3_CL1-Fc fusion protein and FACS analysis. Representative histograms are shown.
(TIF)
