# Supplementary figures and images for: The DRF motif of CXCR6 as chemokine receptor adaptation to adhesion
Source: PLoS One. 2017 Mar 7;12(3):e0173486. doi: 10.1371/journal.pone.0173486 (PMC5340378; doi:10.1371/journal.pone.0173486)

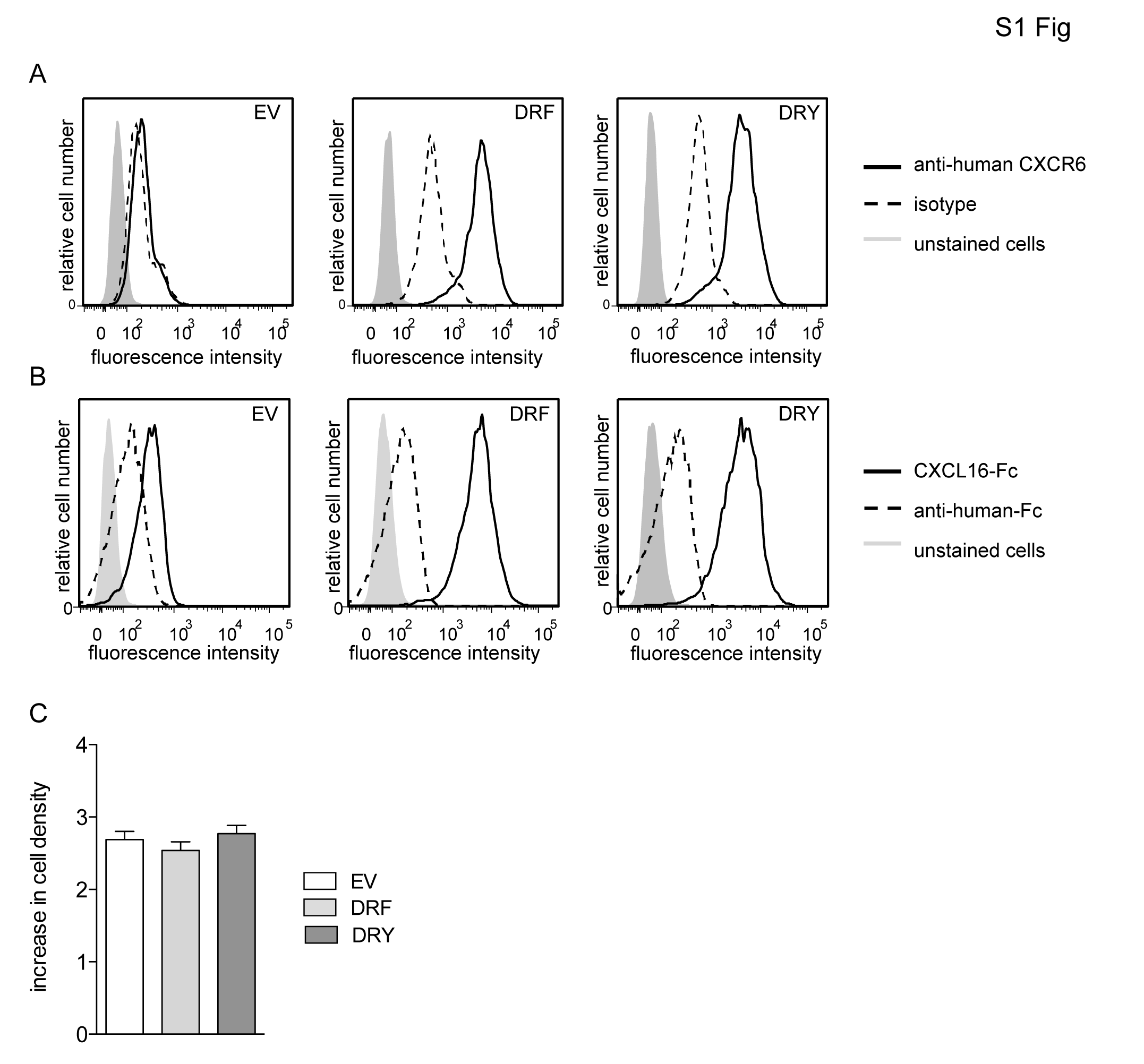

Supplement: S1 Fig — HEK293 cells were transduced with lentivirus encoding human CXCR6 variants or EV control. A: Surface expression of CXCR6 was determined by FACS analysis using an antibody against human CXCR6. Representative histograms are shown. B: Ligand binding was determined by incubation with CXCL16-Fc fusion protein and FACS analysis. Representative histograms are shown. C: Cell proliferation was determined for 72 h by automated real time cell imaging. No statistic differences were observed (n = 3). (TIF) [file pone.0173486.s001.tif]

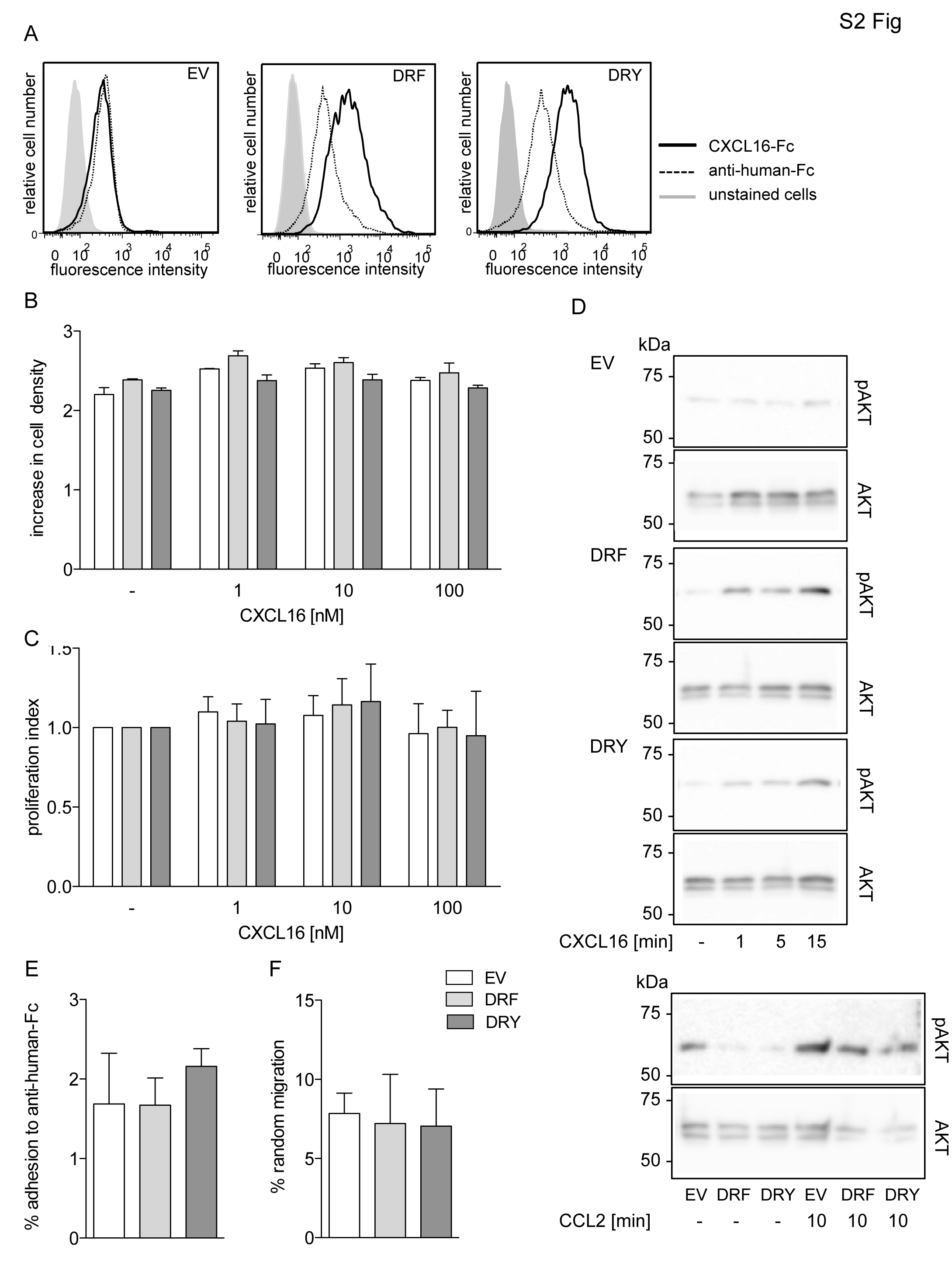

Supplement: S2 Fig — THP-1 cells were transduced with lentivirus encoding human CXCR6 variants or EV control. A: Ligand binding of the CXCL16-Fc fusion protein. Representative histograms are shown. B/C: Cell proliferation in the presence or absence of soluble CXCL16 was determined for 72 h by automated real time cell imaging in B and BrdU assay in C (n = 3). Data in C were expressed in relation to cells in the absence of soluble CXCL16. D: AKT activation was investigated by Western blot analysis. Representative blots are shown. E: Adhesion to immobilized anti-human-Fc was investigated as control experiment (n = 4). F: Random migration was investigated in a Boyden chamber assay (n ≥ 4). No statistic differences were observed in B to E. (TIF) [file pone.0173486.s002.tif]

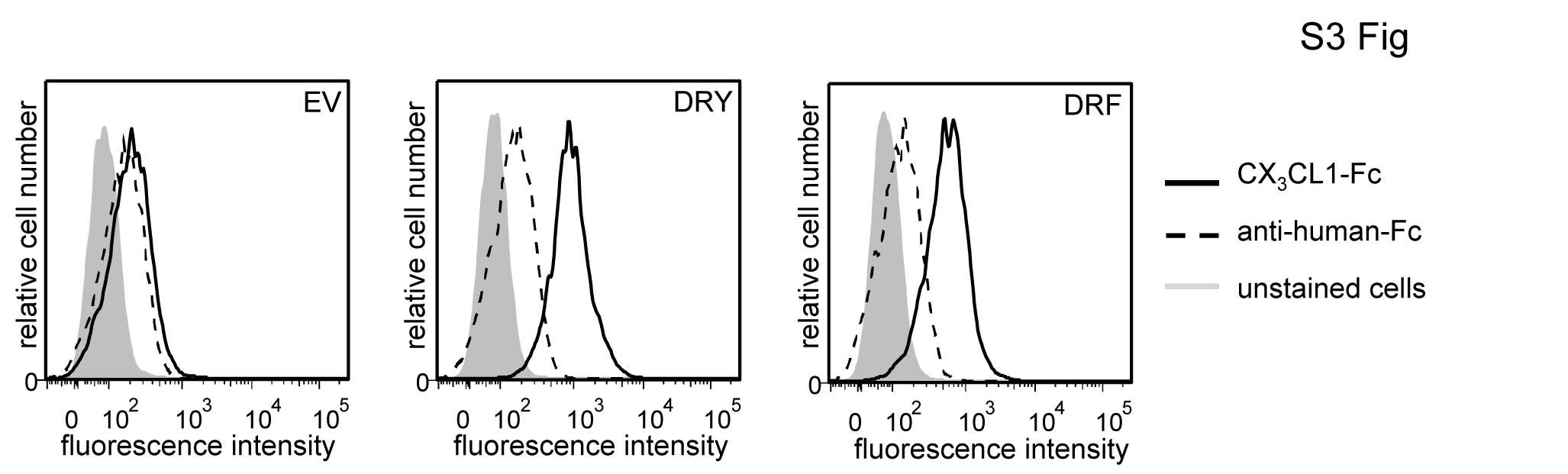

Supplement: S3 Fig — THP-1 cells were transduced with lentivirus encoding murine CX3CR1 variants or EV control. Ligand binding was analyzed by incubation with CX3CL1-Fc fusion protein and FACS analysis. Representative histograms are shown. (TIF) [file pone.0173486.s003.tif]
